# Comparison of analgesic effects of intravenous and intranasal ketorolac in patients with mandibular fracture-A Randomized Clinical Trial

**DOI:** 10.4317/jced.55753

**Published:** 2019-09-01

**Authors:** Javad Yazdani, Reza Khorshidi-Khiavi, Saeed Nezafati, Ali Mortazavi, Farrokh Farhadi, Farhad Nojan, Milad Ghanizadeh

**Affiliations:** 1 Department of Oral and Maxillofacial Surgery, Faculty of Dentistry, Tabriz University of Medical Sciences, Tabriz, Iran; 2 Postgraduate Student, Department of Oral and Maxillofacial Surgery, Faculty of Dentistry, Tabriz University of Medical Sciences, Tabriz, Iran

## Abstract

**Background:**

Similarity of pharmacokinetics of intranasal ketorolac to the intravenous form and other advantages have promoted its application. This study compared the analgesic effects of intravenous and intranasal ketorolac in patients undergoing mandibular fracture surgery.

**Material and Methods:**

In this clinical trial study, Sixty-four patients with unilateral mandibular fracture were divided randomly into two groups. In group 1, 30 mg of intravenous (IV) ketorolac was injected every 8 hours and in group 2, intranasal (IN) ketorolac spray was used as a 100-µL puff in each nostril (31.5 mg) every 6 hours. After each patient regained consciousness, pain intensity was measured based on visual analogue scale for 48 hours. Finally, the total dose of the opioid analgesic agent (pethidine) and the time for the first request for an analgesic agent were recorded for each patient, and their means were compared in each group with proper statistical tests.

**Results:**

Mean pain intensity of patients at baseline was significantly higher than that at other intervals and then, it decreased significantly (*P*<0.001). Furthermore, 2, 4, 6 and 8 hours after surgery, mean pain intensity in the IN group was significantly lower than that in the IV group (*P*<0.05). In the IN group, dose of antinociceptive medicine was slightly higher and the time to request it was shorter than the other group, but it was not statistically significant (*P* >0.05).

**Conclusions:**

Application of intranasal ketorolac spray decreased pain after mandibular fracture surgery, especially at 8-hour interval after surgery, decreasing the need for opioids.

** Key words:**Ketorolac, intranasal, intravenous, mandibular fracture.

## Introduction

Pain which is an unpleasant feeling caused by destruction of a tissue disrupts the daily life even in its lowest rate. One of the most agonizing pains for patients is postoperative pain which is caused by inflammation and release of chemical mediators (1,2). Lack of pain during the postoperative period is one of the necessary factors for discharging patients from the hospital (3,4).

Antinociceptive drugs typically consist of opioids which might have side effects like postoperative ileus, urinary retention, itching, dizziness, dependence, tolerance, addiction, vomiting and nausea. Based on the report of the Commission of Healthcare Accreditation Organization (CAHO), use of opioids in the postoperative period leads to patient dissatisfaction. On the other hand, use of partial agonists like tramadol has side effects like nausea, vomiting and ileus (3-5). Therefore, many researchers and clinicians are seeking non-opioid antinociceptive drugs. Medicines like NSAID and NMDA (N-Methyl-D-Aspartate) agonists, α2 agonists, calcium channel blockers, local infiltration or peripheral neural block with anesthetics, and non-pharmaceutical agents like transcutaneous electrical nerve stimulation (TENS) have been used, each with their own strengths, weaknesses and side effects (5).

Currently, use of NSAIDs, in combination with opioids or alone, is a part of multimodal antinociceptive treatments in controlling mild-to-moderate pains such as pain after maxillofacial, orthopedic and outpatient surgeries (2,6,7).

Ketorolac is an injectable NSAID drug with antinociceptive and antiinflammatory properties. This drug belongs to heterocyclic acetic acid family and has high antinociceptive effects and moderate antiinflammatory properties. The effect of this medicine occurs through blocking the formation of prostaglandins by blocking cyclooxygenase enzyme. Ketorolac, like other NSAIDs, is a non-selective COX inhibitor. Ketorolac is used to manage short-term moderate-to-severe postoperative pains. Maximum allowed time to use this medicine in oral form is 5 days, with 2 days for the injection form (intravenous or intramuscular) (1,8-10).

Intranasal spray (31.5 mg every 6–8 hours for 5 days) is another form of ketorolac, which has been introduced in recent years. Pharmacokinetics and half-life of intranasal spray are similar to the intravenous form. Its application have been promoted by its other advantages like ease of use, lack of pain during its intravenous or intramuscular injection and its use in patients with lockout oral feeding (11).

Previous studies have shown that prescription of intranasal ketorolac reduces pain in patients undergoing impacted tooth surgery (12), dental implants surgery (13) and orthopedic and abdominal surgeries (14). Furthermore, in a study, intranasal ketorolac in the first hour after surgery in 300 candidates for hysterectomy and hip replacement reduced pain compared to the control group. In addition, patients received less opioids (11). He *et al.* (15) in a systematic review in 2012, reported that using intranasal ketorolac spray can significantly reduce postoperative pain of patients. Yet, no comprehensive study is available on the application of intravenous and intranasal ketorolac in patients undergoing maxillofacial fracture surgeries. In most above-mentioned studies, the application of intranasal ketorolac as an antinociceptive agent has been confirmed against placebo. Therefore, this study aimed to compare the antinociceptive effects of intravenous and intranasal ketorolac in mandible fracture patients. If the antinociceptive effects of intranasal ketorolac are equal or better than its injected form, it can replace injected form due to its ease of use.

## Material and Methods

In this parallel randomized clinical trial, conducted from March to September 2018 in Imam Reza Hospital in Tabriz, 64 patients, with unilateral fracture in the body, angle and parasymphysis of the mandible with indication for open reduction with intraoral method, were selected by simple random sampling and included in the study after obtaining informed consent.

-Determining the sample size

The results of a study by Alexander et al(16) were used to determine the sample size, in which the mean pain intensities in the intranasal ketorolac and control groups were 64.5±1.9 and 63.1±1.5, respectively. By considering α=0.05 and 80% study power, 25 samples were selected for each group; however, the sample size increased up to 25% in order to increase the accuracy of the study. Therefore, 32 samples were estimated for each group and totally, 64 subjects were selected.

-Inclusion criteria 

1) Consent and intention to participate in the study

2) Patients with unilateral maxillofacial fracture in the body, angle and parasymphysis, with fracture occurring not more than two weeks ago

3) Patients in an age range of 20–60 years (due to higher incidence of maxillofacial traumas in this age range) (17)

4) ASA I (healthy patients) and ASA II patients (patients with mild systemic problems and without functional limitations (18)

5) A VAS of ≥4 immediately after regaining consciousness (moderate to severe pain) (13)

-Exclusion criteria 

1) Surgery lasting for >2 hours (due to complicated operation)

2) Drug abuse by the patient 

3) Psychopathic patients (all the patients taking psychopathic medicines were excluded due to palliative effects of these medications)

4) Drug allergy 

5) Systemic diseases that might be exacerbated due to drug side effects

6) A history of seizures (due to anticonvulsive drug effects)

7) More than one incision site 

8) Use of more than two plates

9) Edentulous patients (due to the difference in surgery procedure like circum wire)

-Description of study groups 

The patients were divided randomly into two groups by a person not aware of the study objectives by using RandList software. In group 1, a 30-mg dose of intravenous ketorolac every 8 hours was prescribed and in group 2, a 15.75-mg dose of intranasal ketorolac (SPRIX, Egalet US Inc, USA) was prescribed as a 100-µL puff in each nostril (31.5 mg) every 6 hours.

-Evaluation of the study outcomes 

The primary outcome of this study was the relief of postoperative pain. After regaining consciousness, the pain intensity was measured based on VAS for 48 hours at baseline and at 2-, 4-, 6-, 12-, 24-, 36- and 48-hour intervals. For this purpose, the patients were asked to express their pain intensity as a number from 0 (without pain) to 10 (severe and unbearable pain). Mean pain score for each patient in the study groups and at different time intervals were recorded and compared. The physician prescribed pethidine (25 mg) intravenously by the request of patient (PRN). Finally, total opioid dose (pethidine) was recorded for each patient in mg during the first 48 hours and the time of request for antinociceptive in minutes and the means of the groups were compared.

Before initiating the study, each nurse was given the necessary instructions by the researcher about how to fill the checklist. The study process and pain scoring system were explained to the patients. All the patients completed the study and none left the study or was excluded. In this study, the surgeon and nurses in the ward, who recorded the prescribed doses of opioids and the first request for antinociceptives by the patient, were blinded to the study procedures. All the surgeries were carried out by a maxillofacial surgeon.

All the patients underwent the general diagnostic procedures and treatment planning and none was deprived of care. All the ethical aspects of this study were approved by the Ethics Committee of Tabriz University of Medical Sciences (IR.TBZMED.REC.1396.1270). The study was registered in Iranian Clinical Trials Registration Center under the code IRCT2015062802295NB. At the beginning of the study, informed consent forms were signed by the subjects after proper explanations were provided by the researcher and explained to the patients in order to solve the problem of illiterate patients.

-Analysis of data 

Data were analyzed with descriptive statistical methods (frequencies, percentages, means and standard deviations), one-way ANOVA and repeated measures ANOVA using SPSS 19. Data normality was evaluated by Kolmogorov-Smirnov test. Statistical significance was set at *P*<0.05.

## Results

**Figure 1:**
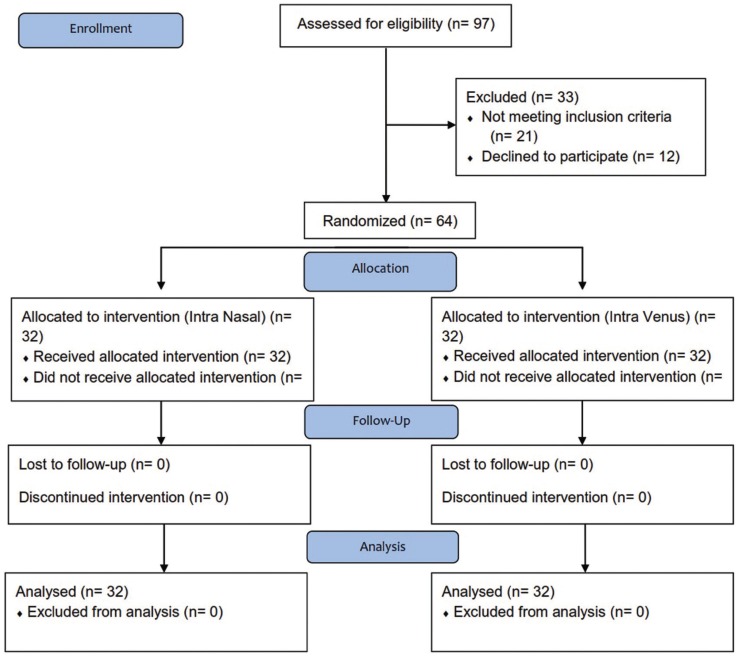
The CONSORT Flow diagram of study patients.

In this study, 64 patients (48 men and 16 women) were evaluated with a mean age of 14.7±33.7 years ( Table 1) (Fig. 1).

**Table 1:**
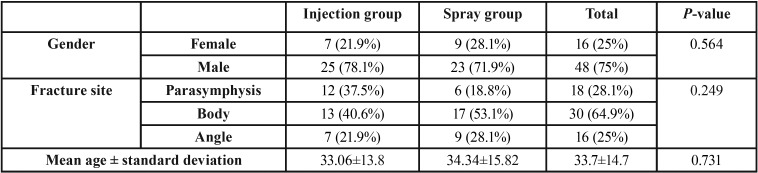
Mean ages and frequencies (percentages) in terms of gender and fracture site of the patients.

The results of chi-squared test in terms of the gender of participants and the fracture site, and independent t-test in terms of mean age of patients showed no significant differences between the study groups (*P*>0.05). In other words, patients in both groups were the same regarding mean age and fracture type.

 Table 2 indicates the mean pain intensity of patients in the study groups at different time intervals. As seen, pain intensity in patients immediately after regaining consciousness was highest, decreasing gradually in 48 hours. Repeated-measures ANOVA showed that this was statistically significant (*P*<0.001). Sidak test was used for pairwise comparisons of different time intervals and mean pain intensity of patients. The results are presented in  Table 3.

**Table 2:**
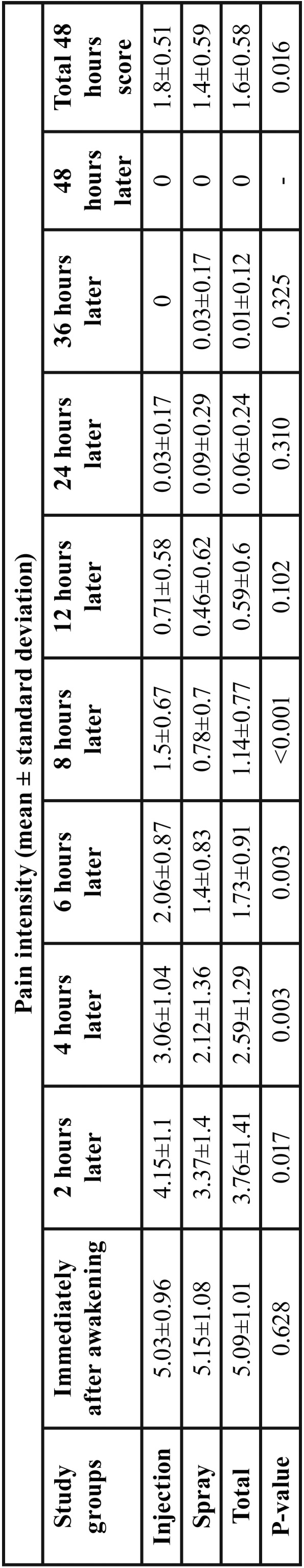
Means ± standard deviations of pain intensity at different time intervals.

**Table 3:**
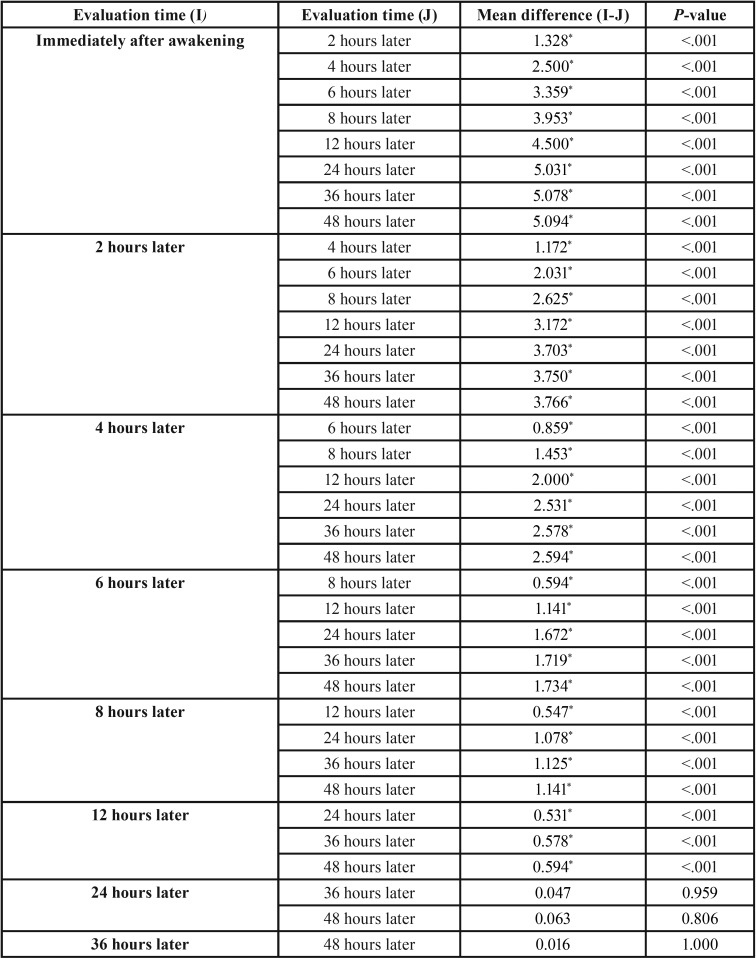
Sidak test results.

Based on Sidak test results, mean pain intensity of patients immediately after regaining consciousness was significantly higher than the means at other intervals and it was at the highest level (*P*<0.001).

Patients’ pain intensity 2 hours after receiving the first antinociceptive dose was the highest compared to 4-, 6-, 8-, 12-, 24-, 36- and 48-hour intervals (*P*<0.001). This process continued 12 hours after prescribing drug such that at each time interval of the study, pain intensity was higher compared to the following time intervals (*P*<0.001). In comparison of mean pain intensity in patients at 24-, 36- and 48-hour intervals after receiving drug, although slight clinical reduction in pain intensity of patients was observed, this decrease was not significant statistically (*P*>0.05). Therefore, mean pain intensity of patients 24 hours after receiving medicine up to 48 hours later was the same and in a very low level.

Independent-samples t-test was used to study the effect of antinociceptive drug on mean pain intensity at each study interval. The results of this test showed that 2, 4, 6 and 8 hours after surgery, mean pain intensity in the ketorolac spray group was significantly lower than that in the injection group. In addition, mean 48-hour mean pain intensity in the ketorolac spray group was significantly lower than that in the other group (*P*<0.05). In contrast, immediately after regaining consciousness and 12, 24, 36 and 48 hours after surgery, means of pain intensity were not significantly different between the two groups (*P*>0.05).

The prescribed dose of antinociceptive agent during the 48-hour period after surgery and the first request for antinociceptive were recorded separately for each group ( Table 4). The results indicated that in the spray group, the dose of the antinociceptive agent was slightly higher and the time to request the antinociceptive agent was slightly shorter than other group. However, t-test for independent groups showed that this was not statistically significant (*P*>0.05).

**Table 4:**
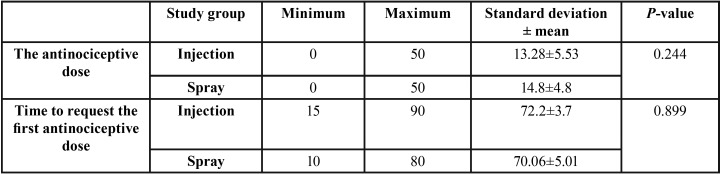
Mean prescribed dose of antinociceptive drug (mg) and the time of first antinociceptive request in different study groups.

## Discussion

Use of opioid antinociceptives results in patients’ dissatisfaction due to their various side effects. Therefore, clinicians are not much interested in using them (3-5). On the contrary, there is more willingness to apply effective compounds to the central and peripheral nervous systems and use multimodal methods to reduce pain severity (19,20).

NSAID drugs have been reported to be effective as a part of antinociceptive multimodal treatments in controlling mild to moderate pains, like maxillofacial postoperative pain, small orthopedic surgeries and outpatient surgeries (6,7). Ketorolac is an NSAID drug and belongs to heterocyclic acetic acid family with high antinociceptive and moderate antiinflammatory properties (9,10).

The results of this study, which was designed and implemented to compare palliative effect of intravenous and intranasal ketorolac in mandible fracture surgeries, indicated that mean pain intensity of patients in both groups immediately after regaining consciousness was highest but it significantly decreased in 48 hours. Patients in the ketorolac spray group exhibited lower pain during the first 2 to 8 hours after regaining consciousness compared to the other group. Since patients in the intranasal ketorolac group received spray every 6 hours and every 8 hours in the injection group, in the first 8 hours, two doses (63 mg) and 1 dose (30 mg) were prescribed in spray and injection groups, respectively. Therefore, receiving a higher dose in the spray group can be effective in the lower pain intensity of patients in this group. Different studies indicated that pharmacokinetics and half-life of intranasal ketorolac (5.24 hours) is similar to its intravenous form (5–6 hours), explaining its considerable antinociceptive effects of intranasal ketorolac (12,21).

In this study, 12 to 48 hours after surgery, pain intensity was the same in both groups but its intensity decreased compared to the first 8 hours. The results also showed that the mean time of first opioid drug request and its dose were the same in both groups.

Brown *et al.* (11) studied 300 candidates of hysterectomy and hip replacement patients who needed at least 5 days of hospitalization. In one group, 100 µL of intranasal ketorolac was prescribed in each nostril every 8 hours in 5 days, and placebo (in the same dose and as intranasal spray) was used in the other group. Patients received morphine sulfate after surgery and this drug was injected upon their request. The results indicated that in the first hour after surgery, intranasal ketorolac significantly reduced the pain intensity. They concluded that rapid pain relief property of intranasal ketorolac can be useful in managing moderate to severe postoperative pain. In addition, patients in the intranasal ketorolac group received less opioids. The results of the study above are consistent with those of the present study, which indicated that intranasal ketorolac in early hours after surgery can significantly reduce the pain intensity without increasing the dose of opioid agents. Of course, in this study, patients in both groups asked for opioids in the first 70 minutes after regaining consciousness and receiving ketorolac injection or spray. However, in a study by Brown *et al.* (11), patients did not ask for opioids at least three hours after surgery. These values are very lower than 6–8-hour interval which has been known as the mean painless time caused by each ketorolac dose (22). This difference in the results can be due to the difference in surgeries and the injury intensity. It is also possible that the reason for early request of opioids was the fact that the patients expected free access to opioids.

He *et al.* (15) reported in a systematic review that using intranasal ketorolac reduces the postoperative pain significantly.

Singla *et al.* (23) showed that prescribing intranasal ketorolac with a 31.5-mg dose 6 times a day in the first 48 hours and 4 times a day for the next 2 to 5 days, reduces the pain and discomfort of patients undergoing abdominal surgery and the need for opioids after surgery.

Grant *et al.* (12) evaluated 80 impacted tooth surgery candidates. Immediately after surgery, 100 µL of intranasal ketorolac was used in each nostril of a group (31.5 mg) and placebo in the other group. Pain intensity was measured 20, 40, 60 and 90 minutes, and 2, 3, 4, 5, 6, 7, and 8 hours after surgery based on VAS. The results showed that in the first 20 minutes after surgery, the antinociceptive effects of ketorolac were high and continued for the next 6–8 hours.

Bockow *et al.* (13) showed that prescribing 31.5 mg of intranasal ketorolac reduced pain in the first 20 minutes after implant surgery and its effects lasted for 6 hours.

These results are consistent with our results that prescribing ketorolac, especially in the intranasal form, had antinociceptive effects 6 to 8 hours after surgery. Of course, in all these studies, the effect of placebo was compared with ketorolac spray. In this study, for the first time, the effects of ketorolac injection and spray were compared.

Previous studies reported side effects for intranasal ketorolac. Approximately 24% of patients who used intranasal ketorolac exhibited transient nasal passage which was removed a few minutes after consuming the drug (11). Headache and burning nasal mucus were reported after using intranasal ketorolac (12,13). In the present study, patients had no problem, and no case of nose inflammation and other effects were reported.

The use of intranasal ketorolac in patients with inter-maxillary fixation and limited oral antinociceptive agents can be useful. The similarity of pharmacokinetics and half-life of intranasal spray in its injected form and other advantages, like ease of use and not imposing intravenous or intramuscular injection pain, have increased the interest in its use. Of course, ketorolac spray is recommended only for 5 days because it has some side effects (11).

-Limitations and suggestions 

There was no control group in this study. Therefore, the accurate and comprehensive comparison of opioid dose and painless time based on antinociceptive medicine was challenged. Although one maxillofacial surgeon conducted all the surgeries, creating the same accurate condition for all the patients, including surgery duration, trauma imposed on the surgery site, fracture type and other psychological factors, was not possible, which might have influenced the results of the study.

On the other hand, it was not possible to blind the patients to the study because some of them received spray and some received injection. Another limitation of this study was the small sample size. It is suggested that larger sample sizes be used in future studies. Simultaneous use of oral or injected acetaminophen with intranasal ketorolac to manage acute pain in the first 6 hours after surgery is another suggestion that can be considered in future studies. In addition, in this study, only intravenous injection was used while other studies believe that intramuscular injection is more effective.
